# Low-Burden Electronic Health Record Strategies for Engaging Oncologists in Digital Health Behavior Change Interventions: Qualitative Interview Study

**DOI:** 10.2196/65975

**Published:** 2025-05-30

**Authors:** Monisola Jayeoba, Courtney L Scherr, Allison J Carroll, Elyse Daly, Savanna Kerstiens, Siobhan M Phillips, Brian Hitsman, Sofia F Garcia, Bonnie Spring, Maia Jacobs

**Affiliations:** 1 Department of Communication Studies School of Communication Northwestern University Evanston, IL United States; 2 Department of Computer Science McCormick School of Engineering and Applied Science Northwestern University Evanston, IL United States; 3 Department of Psychiatry and Behavioral Sciences Feinberg School of Medicine Northwestern University Chicago, IL United States; 4 Department of Preventive Medicine Feinberg School of Medicine Northwestern University Chicago, IL United States; 5 Robert H. Lurie Comprehensive Cancer Center Northwestern University Chicago, IL United States; 6 Department of Medical Social Sciences Feinberg School of Medicine Northwestern University Chicago, IL United States; 7 Department of Behavioral Science and Social Medicine College of Medicine Florida State University Tallahassee, IL United States

**Keywords:** digital health behavior change, electronic health record, risk behavior, human-centered design, cancer survivorship, clinician-patient communication

## Abstract

**Background:**

Digital health behavior change interventions play an important role in helping cancer survivors improve their quality of life and reduce the risk of cancer recurrence. Clinician-patient communication is central to promoting the uptake of and adherence to digital health behavior change interventions. However, oncologists face significant barriers, including time constraints, knowledge gaps, and conversational uneasiness that limit risk behavior and health behavior change conversations.

**Objective:**

This qualitative study aims to explore oncologists’ preferences for discussing and monitoring risk behaviors with cancer survivors, with a specific focus on conversations about digital health behavior change interventions. This study also aims to explore oncologists’ informational and technological support requirements to facilitate these conversations.

**Methods:**

We conducted semistructured interviews with 18 oncologists who provide cancer care in a large National Cancer Institute–designated comprehensive cancer center. The transcripts and interview notes were analyzed through an iterative thematic analysis to generate relevant themes and categories.

**Results:**

We identified 2 major themes with 7 subthemes. The first theme focused on oncologists’ desired roles in promoting health behavior change, while the second theme addressed the support needs to facilitate conversations about risk and health promotion. Oncologists expressed a desire for 2 action-oriented communication mechanisms for promoting digital health behavior change with their patients: referring patients to interventions and reinforcing intervention goals longitudinally. To facilitate risk behavior and health behavior change conversations, their support needs included a preference for low-burden, electronic health record–integrated tools providing timely updates on patient enrollment and progress. The participating oncologists requested a tailored conversation aid for patient communication and parallel systems combining electronic health record messaging with print materials. They also emphasized the need for automated recommender systems to identify and refer eligible patients and reminder systems to prompt timely discussions with patients.

**Conclusions:**

Oncologists are motivated and well-positioned to support patients’ health behavior change but have unmet informational and technological requirements. On the basis of oncologists’ perspectives, our findings provide actionable, user-centered, low-burden strategies for facilitating oncologist-patient conversations about digital health behavior change interventions. We make recommendations for integrating these strategies directly into the electronic medical record system, with the goal of amplifying oncologists’ influential roles in motivating health behavior change among survivors. These scalable strategies may be applicable beyond oncology to clinical contexts where greater promotion of patients’ health behavior change is desired.

## Introduction

### Background

Health behavior change is critical for many cancer survivors, as an estimated 71.1% of survivors who complete the treatment engage in at least 1 prevalent health risk behavior (smoking, insufficient physical activity, and poor diet) [1]. These risk behaviors increase survivors’ susceptibility to cardiovascular diseases, cancer recurrence, reduced quality of life, and increased health care costs [2-5]. To support health behavior change, digital health behavior change interventions, which include behavioral treatments for modifying health risk behaviors [6,7], are becoming increasingly available to patients to promote healthier lifestyles.

Although digital health behavior change interventions have been shown to be helpful in facilitating behavior change [8,9], such as smoking [10-12], physical activity [13,14], and overeating and poor diet [15-20], the long-term adherence remains low [21]. This is partly because such interventions are typically delivered through community programs, research studies, or clinical trials in outpatient settings and often lack integration with patients’ oncology care providers [22]. For digital health interventions to offer sustained improvement to health outcomes, they must be adopted into the health care system and designed to meet the requirements and preferences of patients, health care providers, and clinical context [21-23]. As more studies aim to increase the implementation of health behavior change interventions into health care systems, questions regarding how these interventions should involve clinicians to improve real-world adherence remain. This study explores oncologists’ preferences for low-burden strategies to enhance their involvement in health behavior change intervention studies.

Previous research has consistently shown that clinician-patient communication is central to promoting health behavior change and increasing uptake and adherence to digital health behavior change interventions [24-26]. When clinicians refer patients to behavior change interventions, they provide crucial supportive accountability [27,28] and can increase patients’ self-efficacy in adopting healthier lifestyles [4]. Other studies have suggested that when clinicians actively motivate and support patients’ involvement in health behavior change interventions, it can enhance their understanding and trust in the intervention, potentially leading to long-term adherence [29-31].

However, oncologists also often face time constraints due to high workloads and competing demands, which limit their capacity to discuss risk behaviors and behavior change interventions [32,33]. Insufficient knowledge about behavior change techniques [31,34] and conversational uneasiness driven by the fear of causing offense are other prevalent barriers hindering discussions on health behavior change [35-39]. As digital health interventions become more ubiquitous, there is a need for strategies to overcome the existing communication barriers and improve oncologists’ involvement and clinician-patient communication in patient-facing health behavior change interventions [40,41].

### Objectives

A few studies have attempted to explore clinicians’ perspectives on implementing digital health behavior change interventions [32,42,43]. However, oncologists’ perspectives on overcoming barriers to discussing risk behaviors and promoting these interventions with patients are under-studied. Involving clinicians in creating strategies is crucial to ensure the resources developed are congruent with their needs and the realities of clinical practice in ways that facilitate care delivery and patient engagement [44]. Hence, the aim of this qualitative study is to explore oncologists’ preferences for low-burden strategies to facilitate oncologist-patient communication about risk behaviors and behavior change, with a specific focus on conversations about digital health behavior change interventions. The study also explored oncologists’ informational and technological support requirements to facilitate these conversations. Our findings provide recommendations for developing low-burden strategies that can be seamlessly adopted and sustained in practice.

## Methods

### Overview and Study Design

The study took place in a National Cancer Institute–designated comprehensive cancer center situated within a university-affiliated health care network in the Chicago metropolitan area. Study participants were oncologists (clinicians providing cancer and cancer survivorship care within the cancer center). We conducted one-on-one semistructured interviews with 18 oncologists.

### Sampling and Recruitment

We used purposive sampling methods complemented by snowball sampling to identify and recruit oncologists for the study [45]. Eligibility requirements included (1) oncologists who were currently providing patient care and (2) had at least 1 year of cancer survivorship care experience. On the basis of these selection criteria, we interviewed 18 oncologists with diverse specializations, including breast, gastrointestinal, genitourinary, radiation, lung, cancer genetics, brain tumor, lymphoma, and neurology. Participants, on average, had 15.7 (SD 12.2) years of medical practice experience. Table 1 provides a summary of participants’ demographic data.

**Table 1:** Study participants’ demographic data, the approximate percentage of patients in survivorship care, and years of medical practice (N=18).

Demographic characteristics			Participants, n (%)
**Race**			
	Asian	2 (11)	
	White	14 (78)	
	Other	2 (11)	
Sex			
	Female	10 (56)	
	Male	8 (44)	
Estimated patients in postprimary treatment (%)			
	0-24.9	9 (50)	
	25-49.9	4 (22)	
	50-74.9	3 (17)	
	75-100	2 (11)	
Approximate duration of medical practice (y)			
	0-10	7 (39)	
	11-20	6 (33)	
	21-30	3 (17)	
	31-40	2 (11)	

### Data Collection

**Figure 1:**
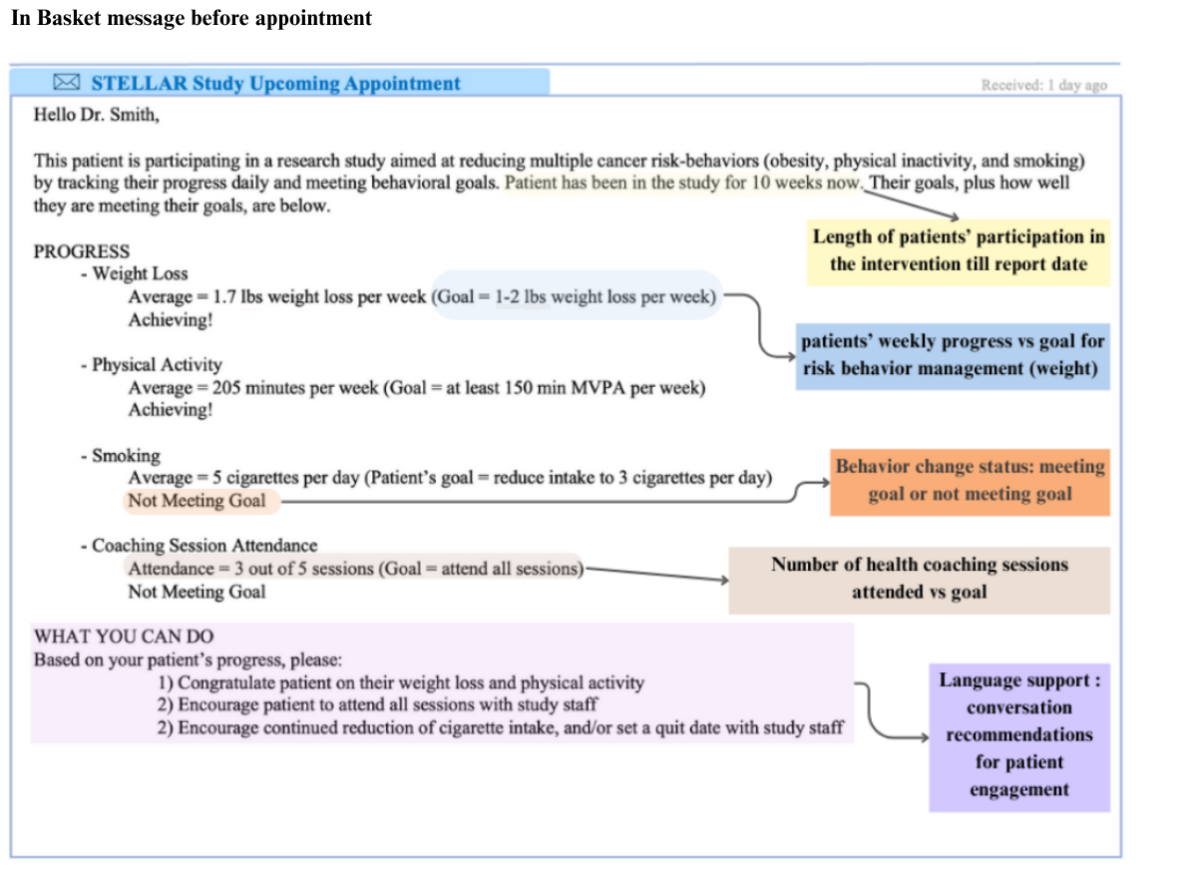
Low-fidelity prototype of sample longitudinal information for a digital health behavior change intervention.

Contextually, after patients complete active primary cancer treatments, they typically have less frequent visits to their oncologists. Oftentimes, the consultations are scheduled 3 to 6 months apart, during which priority is given to follow-up laboratory result reviews, comorbidity conversations, and fewer lifestyle management discussions due to the several barriers mentioned in the Introduction section. Using a semistructured interview guide, we asked participating oncologists about their motivations and deterrents for communicating with cancer survivors about health risk behaviors and behavior change, current approaches, and improvement opportunities. We also talked with the oncologists about the types of information (patient participation and intervention details) they may or may not want to see as a patient progresses through a behavior change intervention. We used semistructured interviews because they offered a flexible, first-hand opportunity to understand participants’ perspectives and needs [45-47]. After oncologists shared their initial preferences for low-burden strategies to facilitate oncologist-patient communication about risk behaviors and behavior change, we shared a low-fidelity prototype of the type of longitudinal information that could be collected and shared from digital health behavior change interventions. The prototype contained information such as patient goals, behavioral data (such as physical activity data), and conversation assistance (Figure 1). The prototype was obtained from an ongoing pilot study on scalable telemedicine for cancer survivors [22]. We included this prototype as this visual elicitation method has been shown to be a useful method for engaging participants in thinking about future solutions [48]. The objective was to gather insights into crucial information from the oncologists’ vantage, optimal content organization, and effective communication.

One-on-one semistructured interviews were scheduled for 1 hour and conducted via Zoom (Zoom Communications, Inc) conferencing with video communication enabled. All the interview sessions were recorded. Transcripts were generated via Zoom and reviewed by a research team member for accuracy. The team members also ensured that transcripts were completely anonymized and did not contain participants’ identifiable data. Participants provided their demographic information during the interview sessions.

### Data Analysis

The completed interview transcripts served as the basis for the data analysis. We analyzed the transcript data following the 6 steps for thematic qualitative data analysis proposed by Braun and Clarke [49]. First, we immersed ourselves in the data to gain an in-depth understanding. Then, the preliminary analysis or open coding commenced concurrently with ongoing data collection, during which we identified emerging themes. The themes were drawn inductively with regard to the study’s research questions, and the codes were interpreted at the semantic level. We performed the second coding round with MaxQDA software [50], complemented with Miro for sorting and categorizing the themes. We stopped new data collection when we reached data saturation and no longer found new themes [51]. In both coding rounds, 2 research team members recursively evaluated the data, using constant comparison to examine themes, reduce irregularities, and establish patterns across data and themes, to achieve congruence.

### Ethical Considerations

Ethics approval was obtained from the Northwestern University’s institutional review board (STU00217509). Before the interviews, participants were emailed a copy of the consent form to review. During the remote interviews, the interviewers reviewed the consent form with participants, after which participants provided oral consent.

To ensure the privacy and confidentiality protection of our study participants, the research team members removed all the participants’ identifiable data, that is, names, contact details, and other identifiers, during data transcription before data analysis. We replaced participants’ personally identifiable information with coded identifiers (eg, P1 and P2). Data were securely stored on university servers, and access was limited to authorized research team members. Interview sessions were conducted between August 2023 and November 2023, and the study participants were each given US $50 compensation via an electronic gift card. Finally, we followed the standards for reporting qualitative research [52] to ensure transparency throughout the research process.

## Results

### Overview

All the participating oncologists agreed that digital health behavior change interventions are important due to their ability to improve cancer survivors’ quality of life after cancer care. They believed their patients would be interested in using such interventions, as patients often inquired about them. The oncologists shared several ways they would like to engage patients in addressing risk behaviors and discussing health behavior change interventions. However, oncologists also discussed that common barriers, such as a lack of time and limited expert knowledge of health behavior change, limit their efforts to hold those conversations with patients. For example, a participant said the following:

I don’t think I have personally enough training or knowledge to know to give them specific recommendations on what they should do. And I can tell they want to know. ...So I think it’s a lack of time in clinic, and a lack of probably knowledge from my end on how to counsel them.

P5

Hence, they discussed the support requirements that could help overcome those barriers and enhance their conversations with patients on these topics. We elaborate on our findings in the subsequent subsections.

### Findings

Our findings are broadly categorized into two main themes describing clinicians’ preferences for engaging with patients in health behavior change interventions: (1) oncologists’ desired roles in promoting health behavior change interventions and (2) support needs to facilitate risk behavior and health promotion conversations. Textbox 1 gives the highlights of the main themes and subthemes.

**Themes and subthemes that capture low-burden strategies for engaging clinicians in patient digital health behavior change interventions.**

**Theme 1: oncologists’ desired roles in promoting health behavior change interventions**

- Referring patients to relevant health behavior change interventions (community programs, research studies, or clinical trials)
- Reinforcing intervention goals longitudinally

**Theme 2: support needs to facilitate risk and health promotion conversations**

- Information needs: intervention details; patient enrollment information; and medium, modality, and recipient preferences
- Overlapping and parallel systems to facilitate easy and time-efficient conversations: electronic health record outbound messages with complementary print materials, a reminder and recommender system for just-in-time support, and patient progress reports

### Theme 1: Oncologists’ Desired Roles and Barriers in Promoting Health Behavior Change Interventions

Oncologists reported an interest in referring patients to relevant health promotion interventions. Second, they wanted to reinforce patients’ behavioral goals over time.

#### Referring Patients to Relevant Interventions and Studies

Most (17/18, 94%) of the participants explicitly mentioned that they would be happy to refer patients to health behavior change research studies and clinical trials. The clinicians acknowledged that their patients often sought guidance on these interventions, recognizing their potential to help patients address high-risk factors, such as smoking and obesity. Two oncologists stated the following:

I would definitely highly recommend programs like this [digital health behavior change intervention studies]. It’s just so important to be able to try to get patients to, you know, improve those high-risk factors...It’s me and my PRN [pro re nata nurse] who see patients. I think we would both be definitely interested in referring patients.

P7

I easily could see us being a referral source to try to get patients enrolled in the program [health behavior change interventions].

P11

Participants also believed that patients would be more likely to enroll in health behavior change research studies and clinical trials if someone on their care team referred them. They posited that patients will likely find the intervention more trustworthy when discussed with a trusted professional with whom they have built a relationship during their active treatment period. Participants were willing to leverage their position and relationship to refer patients:

We have that relationship with the patient. That’s probably a good segue rather than just some kind of random, unknown person calling them up.

P15

However, based on their previous experiences, some clinicians mentioned concerns about forgetting that behavior change intervention studies exist, forgetting to refer patients and having difficulty identifying eligible patients for the programs:

I would be happy to refer people. I think one of the challenges [is that] oncologists readily think about therapeutic clinical trials, but when it comes to some of these other types of trials that are important, they’re just not in mind, you know...Yeah, it [referring] becomes hard because you’d have to know which individuals are approaching a surveillance-type program.

P2

Participants consistently expressed willingness to refer patients to health behavior intervention studies, recognizing the importance of addressing high-risk factors for improved patient outcomes. They acknowledged their influential role in patient enrollment, citing their established relationships and patients’ trust in their recommendations. However, challenges, such as forgetting about the studies’ existence and difficulty in identifying eligible patients, were noted, indicating areas for potential improvement in facilitating referrals and enhancing clinician engagement with health promotion programs.

#### Reinforcing Intervention Goals Longitudinally

All (18/18, 100%) clinicians expressed interest in encouraging patients’ continued engagement and reinforcing intervention goals. Some participants mentioned that they intended to leverage their preexisting clinician-patient relationship to help patients adhere to the intervention by motivating, encouraging, congratulating, and addressing noncompliance when relevant:

I think if I’m sure there’s going to be areas that patients are struggling with...knowing that [information] ahead of time, that’s something that we can also probably reinforce in clinic when we see the patients.

P5

Some clinicians suggested that patients could greatly benefit from the intervention through reinforcement, as patients may perceive clinicians as holding them accountable, potentially enhancing adherence:

If you’re not meeting some measure of success, we can intervene. You know, after a month, there’s been zero weight loss, they’re smoking more than they were before. We could chime in and say, hey, love that you’re participating in this study. Just want to reinforce. I think it’s a great idea.

P3

However, most clinicians raised concerns about the limited time they spent with patients during their infrequent appointments and the lack of expert knowledge on behavior change. They expressed how these factors constrained their ability to accommodate the extra workload of holding behavior change conversations with their patients. One participant shared the following:

I think, completely agree with the lack of time that we have to talk about this with patients, because we have often just like a 20-minute slot...I know patients are interested in learning more, and I also think, aside from me, saying yes, stay active [and] walk. I don’t think I have personally enough training or knowledge to know to give them specific recommendations on what they should do. And I can tell they want to know...So I think it’s a lack of time in clinic and a lack of probably knowledge from my end on how to counsel them.

P5

Participating oncologists indicated interest in encouraging patients and reinforcing their intervention goals, but they experienced well-known barriers such as time and a lack of expert knowledge of behavior change interventions that limited their engagement with patients.

### Theme 2: Support Needs to Facilitate Risk and Health Promotion Conversations

To support their desired roles in promoting patient behavior change interventions, all the oncologists expressed interest in receiving technologically enabled support to enhance their engagement when discussing risk and health promotion with patients.

#### Information Needs

The clinicians highlighted the need for crucial information to bolster their engagement when referring patients to relevant behavior change interventions and reinforcing patients’ goals. In the subsequent sections, we enumerate the information they require and their preference for receiving the information.

#### Intervention Details

Most clinicians articulated interest in receiving detailed information about available health behavior change intervention studies, including eligibility criteria, intervention timeline, components of intended treatment, expected patient outcome, and intervention point of contact. They perceived that having this information readily available would significantly improve their ability to discuss and refer patients to appropriate interventions. Clinicians emphasized that being well-informed would increase their knowledge and alleviate conversational uneasiness, enabling them to effectively address patients’ queries and encourage participation in these programs. Furthermore, they highlighted the need for integrating health promotion information into existing clinical workflow, suggesting that information should be sent by the intervention team and should be readily accessible to the oncologists just before the patient consultations. According to the oncologists, this support would enhance patient engagement and facilitate successful enrollment and follow-up in health behavior change intervention studies:

We are most successful if everything happens pretty organically. So that, you know, patient comes in for that particular visit. I’m prepared with the information...And having the physician have that information and introduce it as a concept also, I think, really increases the interest.

P14

Oncologists also believed that sharing intervention details, such as components of intended treatment and expected patient outcomes, could support patients’ decision-making process. According to the oncologists, this information increases the likelihood of successfully enrolling patients into recommended health behavior change studies and adhering to the intervention:

And again, I do think the little section on what the evidence is behind it and how it affects cancer outcomes, or could potentially even, you know, sell it even better for patients.

P5

#### Patient Enrollment Information

Most (17/18, 94%) of the participating clinicians stated that they would be interested in receiving enrollment notifications when their patients enroll in health behavior change intervention studies.

Most participants perceived that enrollment information and notifications would create awareness about patient participation, thereby enabling clinicians to reinforce intervention goals more effectively and facilitate treatment adherence:

Yeah, it would be good to know...I think if we know about it. And just kind of like documenting it, making notes when they come back for surveillance, and also just kind of mentioning it to the patients. I think it can also help with compliance and motivate patients to continue.

P7

Participating oncologists showed interest in receiving enrollment notifications. In the subsequent sections, we discuss in depth how clinicians envisioned this desired information being integrated into cancer care to address challenges related to time and information overload.

#### Information Sharing: Medium, Modality, and Recipient Preferences

In addition to identifying the critical information they need, the clinicians discussed how to best convey the information to cancer survivors. Most of the clinicians emphasized that they already face time constraints and receive a high inflow of messages. Therefore, they would prefer an information-sharing and notification system that considers those constraints, particularly in terms of how and to whom information is shared.

Most participants indicated a preference for receiving enrollment notifications via the electronic health record (EHR). In total, 83% (15/18) of the clinicians indicated a preference for receiving the notification via the Epic In-Basket within the EHR; 11% (2/18) of the participants preferred email notifications to their work email addresses. One said either Epic or email works for them. The benefit of driving the In-Basket preference was that it could be viewed by the entire clinical team:

I think Epic In-Basket is probably the easiest...because that could then be forwarded to the team. And so everybody is just aware.

P7

In addition, clinicians shared their preferences for information delivery modality and relevant recipients of the information. In total, 89% (16/18) of the participants noted that a brief text-based notification that indicates patients’ enrollment meets their needs. The rest 11% (2/18) of them mentioned that they would prefer to see a visual banner, that is, a static indicator or pop-up, at the top of a patient’s EHR profile that could lead clinicians to the patient enrollment and participation details within the EHR. They indicated that anytime they visited the patient’s profile, the banner would serve as a notification and continuous reminder of the patient’s enrollment so that they could discuss and reinforce the intervention goals during the visit:

Or if there was a little banner on it like that...[indicating] the patient was participating in a clinical trial. And which one it was.

P12

The clinicians remarked that using a visual banner would reduce distractions from the high influx of messages.

Finally, the clinicians emphasized the importance of sharing information with other members of patient care teams to facilitate collaborative care. All clinicians mentioned that the nurses (nurse practitioners and navigators included) should be aware of patients’ enrollment. They tied this importance to their busy schedules so that patients can get support and reinforcement from other care team members irrespective of the clinicians’ schedule, and the team can collaboratively spread the care workload:

So the nurse would be, it would be helpful. So there’s a decent chance that if I forget, the nurse may remember, or vice versa, and then they might do some of these same tasks. That may be beneficial in, you know, in bringing up to the patients. And then again, it also helps them [nurses] stay aware that they [patients] are on the study. If they [nurses] are getting queries...because oftentimes [patients] will be needing information.

P9

#### Overlapping and Parallel Systems to Facilitate Easy and Time-Efficient Conversations

##### Overview

In addition to information needs, clinicians indicated and discussed their interest in adopting and using technological support parallel to their traditional or existing methods for referring patients and reinforcing intervention goals. As discussed subsequently, participating oncologists suggested that parallel systems like outbound EHR messages with print materials, reminders, and recommendation systems could significantly enhance oncologist-patient engagement:

In terms of us just reaching out to them with a referral, you know I’m old fashioned. If you’ve got a flyer out there, and I have a periodic reminder. I’m going to be most successful in getting that done.

P14

##### EHR Messages With Complementary Print Materials for Patient Referrals

Most clinicians preferred easy and quick methods to refer patients to behavior change interventions. In particular, the clinicians highlighted sending referral messages to eligible patients through the EHR patient portal as an effective means of reaching eligible patients because the messaging system would allow them and their patients to ask questions or seek additional clarification regarding their studies. They encouraged intervention providers to incorporate messages through the EHR patient portal, as they believed those resources would be easy to use and time and energy efficient. In addition, the clinicians showed interest in sharing referral information with their patients through print flyers, as they believed it would aid their conversation with patients and patients’ decision-making:

But, like, you know, it’s a kind of a comprehensive description so that, like, I know about it, and my nurse practitioner, and my nurse, so that if patients are asking them about it, and if it’s, you know, either the flyer or you know some order that can go into Epic. I don’t know if that’s the way. Or, you know, some easy method of referral so that...we can do it quickly and give the information to the patients easily.

P1

Referencing their time constraints, clinicians showed a high interest in resources such as EHR messages to patients and hand flyers as seamless methods to refer patients to the intervention.

##### Reminder and Recommender System for Patient Support

Clinicians stated that they often forget to discuss risk behaviors and health promotion with patients due to the many other competing goals during appointments. To ameliorate this challenge, clinicians expressed interest in technologically enabled systems that would serve as both a recommender and a reminder system. Clinicians wanted a recommender system that would identify if their patient was eligible for any health promotion intervention studies. The reminder system would provide clinicians with just-in-time nudges before meeting the patient to discuss possible interventions and trials with patients. As described by one participant, this type of system would be particularly beneficial when cancer survivors complete active cancer treatment and move to surveillance:

What’s tricky here is to know when a patient may be potentially eligible. So, a pop-up would be ideal...So you know how to identify those people to make it happen.

P2

##### Technological Support for Reinforcing Patients’ Goals Longitudinally: Patient Progress Reports

##### Overview

Every clinician expressed strong interest in reinforcing health behavior change goals over time and expressed interest in receiving simple and just-in-time patient progress reports to enable them to reinforce intervention goals. Referencing their competing clinical priorities and time and staff constraints, the participating oncologists described how the report could be best designed for their optimal and beneficial use regarding features, format, modality, notification medium, and notification frequency.

##### Features and Format of the Report

Clinicians strongly emphasized the need for less time-consuming resources and tools. All clinicians emphasized that the patient progress report should be minimal, concise, readable, and easy to interpret, with only the most relevant information and conversation recommendations. One participant explained the following:

I think we all really want this for all of our patients and for ourselves. But there is a lot of messages, results, and communication we get and it is too much. And if it doesn’t impact my decision making it falls way down on the priority list. So it’ll be important that this communication feels minimal, but a lot of information gets passed along.

P8

Most of the clinicians also indicated a preference for receiving the report through the EHR to ensure easy access and retrieval:

I might just as easily send a note to [reinforce goals] and so that’s why connecting in Epic would be so great.

P13

In terms of modality, most clinicians preferred simple text-based reports that gave a quick and easy-to-interpret overview of patients’ participation. While graphs were appreciated, text-only In-Basket messages provided a highly preferable resource for all participants:

If it’s an Epic [In-Basket message], then we can all see it...and it’s just easy to follow. So anytime, you know, as part of an office visit, we’re going to be reviewing the patient’s chart and looking in to see what has been happening with the patient. So summary information with regards to their progress or their check-ins...That’s helpful because that’s one place we always are in the medical record.

P7

##### Progress Report Details

Given the clinicians’ emphasis on short and easy-to-read reports, we asked participants to share the patients’ progress details they saw as most important. Participants identified a short list of critical elements, which included patients’ behavioral goals, type of behavioral treatment received, frequency of behavioral treatment, and patients’ current behavioral state. Most clinicians highlighted that knowledge of these details would enable them to reinforce patient and intervention goals. They also stated that receiving recommendations or action points for discussing goals, progress, and challenges would aid their conversations with patients:

I like to know what they have achieved and what the goal was. Yeah, not just a summary of if they’re achieving. But yeah, to be more specific about how to congratulate them.

P3

Most clinicians also expressed high interest in knowing the overall patient goal, their immediate or short-term goal at certain times, and the duration of patient engagement with health promotion programs:

But one other thing I would like to know is how long have they been achieving that goal? Is it recent, or has it been for a certain amount of time...So then you know that they’re 10 weeks there, they still have a lot of time. No, that makes sense. I think that that makes sense if we know that they’re going to be in there for a whole year than 10 weeks.

P8

This understanding can be especially important for tailoring follow-up strategies and reinforcement to help patients stay on track or adjust their plans according to how long they have been engaged and how much time remains to meet their objectives. With this information, clinicians are better equipped to provide targeted guidance and support, enhancing the effectiveness of health promotion programs.

Overall, the study participants valued receiving a summary of patients’ progress reports and recommendations for conversations over the raw data driving the progress summary. For example, when asked about receiving raw sensor data from patients’ Wi-Fi scales or smartwatches, most clinicians preferred aggregated data and did not wish to receive raw sensor data:

I guess, right off the bat. I’d probably want more summarized data.

P3

##### Frequency of Notification

All clinicians strongly preferred just-in-time reports of patients’ behavioral changes and health promotion goals that closely aligned with their upcoming appointments. According to the participating oncologists, this approach could prompt and facilitate clinicians’ preparation and allow them to integrate the intervention data effectively into patient interactions:

If we could somehow connect this information to the upcoming patient’s appointment, very fantastic, because then we’d have that lovely data to go over with them.

P14

If this [patient’s behavioral report] is given to me, send it to me, you know, right before I see a patient, it’s easier for me to remember and put it down in my notes that I should bring it up at the next visit...So again, I think the day before, 2 days before while we’re prepping for a clinic, would be the best time to remember, and then we can write a quick note or a note to ourselves to mention it to patients.

P5

The clinicians noted that not receiving the report just before seeing patients may cause underuse of the behavioral change report, as the information might be stored away, perhaps not easily accessed, necessitating some search and retrieval processes, which might be time consuming:

I don’t know if it’ll always be utilized,...if we get the information and we see them 3 months later, or 2 months later, like may or may not. So again. That’s something that we have to figure out on the clinical side...

P7

In addition to just-in-time reports, several clinicians showed interest in getting notified when their patients drop out, discontinue, or complete participation in an intervention or health promotion program:

If there is [provide] a progress note at the end of the [intervention program] study for the patient or whenever the patient finishes [completes the intervention program]. So, if there are people who withdraw early for whatever reason, that there’s kind of at least a brief summary, [showing] the patient participated. They withdrew for this reason, which might be, [that] they completed the study.

P2

Some of them also asked for the progress report to include notes when patients attained substantial progress and outstanding adherence (ie, when patients met outcome parameters such as losing weight as set in their goals) as well as no substantial progress and nonadherence. The clinicians aimed to use knowledge of the notification to congratulate, motivate, and encourage patients according to their outcomes. Overall, the participants saw the just-in-time report received 2 to 3 days before patients’ visits as a crucial enhancement to clinicians’ ability to manage and support patients effectively. It aligned with their routine of reviewing upcoming appointments, allowing sufficient time to assess patient progress and be well informed to hold meaningful discussions during the visits.

## Discussion

### Principal Findings

Our study explored two pivotal roles oncologists want to play in promoting and encouraging health behavior change: (1) referring patients to relevant health behavior change interventions and (2) reinforcing patients’ goals longitudinally. We advance the literature by identifying informational and technological support requirements that oncologists prefer so that they may effectively promote health behavior change with their patients. Clinicians emphasized the need for these solutions to be low burden and seamlessly integrated into their existing practices. They favored easy-to-access intervention details, consolidated just-in-time updates with conversational aids (ie, delivering patient participation updates close to patient visits and including recommendations for supportive conversations), and text-based communication via EHR shared with the entire care team to ensure collaborative care delivery. These low-burden strategies can help overcome the existing communication barriers and enhance oncologists’ involvement in patient-facing health behavior change interventions toward achieving healthier cancer survivorship.

### Oncologists’ Preferences in Digital Health Behavior Change Interventions

#### Overview

While previous research has established the benefits of behavior change interventions for cancer survivors [8,21,53] and the role of oncology care providers in promoting these changes [54], few studies have captured oncologists’ desired roles in supporting patient-facing health behavior change interventions. Findings from our qualitative study address this gap by elucidating 2 action-oriented communication mechanisms through which clinicians want to engage with patients in addressing risk behavior and promoting health behavior change interventions. First, the oncologists in our study expressed a strong motivation to refer their patients to relevant behavior change interventions or health promotion programs. Second, clinicians wanted to provide ongoing reinforcement of patients’ behavior change goals. Despite expressing high interest in referring patients to relevant health behavior change studies and reinforcing intervention goals over time, oncologists face well-known barriers, such as time constraints, the lack of expert knowledge on behavior change, and difficulties identifying eligible patients. To address these challenges, oncologists articulated specific informational and technological support needs. Subsequently, we further discuss how oncologists intend to perform these actions and the support they require throughout a health behavior change intervention life cycle.

#### Patient Recruitment: Identifying Eligible Patients and Making Referrals

Clinicians viewed themselves as well positioned to refer patients to suitable health behavior change interventions and facilitate patient enrollment, given their established relationships and patients’ trust in their recommendations. However, consistent with previous literature, they also noted several challenges, including time constraints [32,33], knowledge gaps [31,34], conversational uneasiness, difficulties remembering available programs, and difficulties identifying eligible patients, which limit their ability to discuss risk behaviors and health promotion interventions in-depth with patients [35-39]. To address these challenges, oncologists requested readily accessible intervention study details, such as eligibility criteria, intervention timeline, treatment components, and expected patient outcomes. This information could help mitigate the knowledge gaps and time constraints hindering clinicians’ referral practices. The oncologists also expressed an interest in automated recommender systems to identify and refer eligible patients to relevant interventions and reminder tools to prompt referral discussions during clinical encounters.

The potential efficacy of EHR-driven clinician reminder and referral systems has been acknowledged to improve specialist referral to intervention services in cancer care [55-59]. In general, the literature has also demonstrated that algorithm-based recommender systems [60-62] within the EHR [57,63] can effectively save time and facilitate patient referrals by delivering personalized, evidence-based recommendations to support clinicians’ decisions during patient care [64-68]. We contribute to the literature recommendations for crucial information that oncologists want to receive from these systems and low-burden strategies that could potentially facilitate integrating these systems into oncologists’ existing clinical workflow.

Although all the study participants were interested in technological support, some were concerned about the possibility of information overload and other increased workloads arising from adopting new digital health technologies [69,70]. Notably, the clinicians preferred parallel communication channels that complement rather than compete with their existing clinical practices. For example, they suggested combining EHR messaging with supplementary print materials (eg, hand flyers) for patient referrals, highlighting oncologists’ interest in using further electronic tools but wariness toward increased EHR interaction burdens. This parallel approach aligns with evidence that EHR-based messaging can improve health behaviors such as cancer screening uptake among patients [71]. We add to this literature the recommendation to include parallel systems for clinicians to share intervention information with patients. These low-burden supports could be pivotal in mitigating the lack of expert knowledge, limited time, and referral pathway barriers that limit clinician-patient conversations on risk behavior and health behavior change interventions.

#### Patient Participation: Following Patients’ Participation and Reinforcing Patients’ Goals Longitudinally

When patients enroll and are actively receiving intervention treatment, oncologists want to play an active role in reinforcing patients’ behavior change goals and supporting their adherence. Clinicians saw themselves and their care teams as crucial sources of motivation, encouragement, and accountability to support patients’ successful adoption of healthier lifestyles. This self-identified reinforcement function in helping patients understand risk factors and facilitating the adoption of risk-averse lifestyles aligns with existing works that have highlighted motivation [29,31] and accountability [27,28] as pathways through which clinician–patient communication contributes to improved health outcomes.

Our findings highlight the potential of EHR-based reports to enhance clinician engagement in providing ongoing reinforcement support. To facilitate their reinforcement function, oncologists desired enrollment notifications and consolidated progress reports integrated into their EHR workflows. They emphasized the need for text-based, just-in-time updates on patients’ behavioral goals and data summaries aligned with patient visits, information that could be quickly reviewed and discussed during upcoming appointments.

Another novel contribution of this study is the identification of clinicians’ need for specific language and conversation recommendations to discuss risk behaviors and health behavior change with patients effectively. This highlights an opportunity to enhance patient–health care provider communication by providing oncologists with clear, concise phrases to explain interventions and their benefits. Participants also stressed the importance of delivering the patients’ behavioral updates to the entire care team, enabling collaborative care and ensuring patients receive consistent reinforcement from various health care providers, even when clinicians’ schedules are constrained.

The clinicians’ need for EHR-based reports to enhance their reinforcement function corroborates evidence in the literature that highlighted the potential of EHR-integrated tools for improving patient–health care provider communication and treatment adherence [72]. We contribute to the existing knowledge by identifying the crucial information oncologists require to reinforce patients’ behavioral change goals: enrollment status, consolidated just-in-time progress reports, discontinuation alerts (dropout and end of program), and tailored conversation aids.

In addition to using the EHR to receive patients’ updates, the oncologists suggested combining EHR messaging (sending messages to the patient portal) with supplementary print materials to provide patients with continual touchpoints and reinforcement outside brief consultation periods. They also highlighted the value of reminder systems for timely discussions about patients’ progress and challenges. By integrating these tools into their EHR, clinicians could access relevant information, and conversation prompts just before seeing patients and outside clinical visits, ensuring meaningful discussions and effective reinforcement of health behavior change goals.

While this study emphasizes EHR-integrated tools for facilitating oncologists’ involvement, it also highlights the importance of complementary communication strategies outside the EHR, such as print materials, for easy referrals. This corroborates previous research that has discussed the relevance of complementary strategies outside electronic messaging, such as holding recurrent team meetings and distributing printed, shared care protocols among patients’ care teams [43]. A key benefit of using this parallel system, that is, integrating digital (EHR messaging) with nondigital (print materials) communication channels, is that it could enhance rapid information exchange and interpersonal interactions while building trust [73], fostering deeper discussions, and maintaining interpersonal care delivery [74]. Adopting this parallel approach can further enhance team performance, potentially contributing to better patient-centered outcomes and coordinated care across different health care settings and networks.

Overall, the study participants’ emphasis on low-burden strategies aligns with previous literature that emphasized the importance of upfront clinical workflow analysis [75-77] and human-centered design for successfully implementing digital health tools and interventions [78,79]. Hence, a unique strength of this qualitative inquiry is that it directly captures the articulated needs and perspectives of oncologists themselves.

### Summary of Recommendations

Through a user-centered approach oriented around clinicians’ lived experiences and workflow realities, we comprehensively gathered their low-burden requirements across information needs, communication preferences, and technological facilitation [80]. The findings provide a road map for developing solutions that can be seamlessly adopted and sustained in practice. On the basis of these findings, we propose recommendations for designing digital health solutions to support oncologists’ involvement in health behavior change intervention studies, as presented in Table 2.

These actionable recommendations address key barriers while leveraging oncologists’ desire to support patient health behavior change, positioning future interventions for more successful real-world implementation and sustainable impacts in cancer survivorship care. While these strategies were developed with oncologists, they are not specifically tied to oncology care. We anticipate that these strategies, given their low resource and low burden characteristics, may be generalized to other health care professionals, such as primary care physicians (PCPs), who also provide important care to cancer survivors.

**Table 2:** Summary of scalable recommendations for designing digital health solutions to support oncologists’ involvement in health behavior change intervention studies.

Time period	Recommendations
Before patient recruitment	Provide clinical teams with intervention study details (eligibility criteria, timeline, treatment components, point of contact, and expected health outcomes) Make information available in EHRa and on print materials
During patient recruitment	Automatically identify eligible patients Include reminder features to prompt referral discussions during clinical encounters Create parallel referral channels by combining automated EHR messaging with supplementary print materials (eg, hand flyers) Send an enrollment notification to the clinical team when patients sign up for the intervention program or trial
During the patients’ intervention use	Send progress reports through the EHR to patients’ clinical team using short, text-based messages Provide aggregated data summaries of patients’ behavioral data (including goals, adherence, and progress) Include a conversation aid: clear and concise phrases tailored to whether the patient is meeting goals (congratulations) or not meeting goals (encouragement) Automate the delivery of patients’ progress report 1 to 2 days before the clinical encounter

^a^EHR: electronic health record.

### Comparison With Prior Work

Our study contributes novel insights and extends 3 previous research studies that have attempted to explore clinicians’ perspectives, including limited oncologists’ perspectives, in supporting health behavior change interventions [32,42,43]. While these previous works have explored this topic, our study addresses significant gaps by focusing specifically on oncologists’ perspectives and needs, which were understudied in previous research. The study by Mylonopoulou [42] examined health care professionals’ views on designing supportive technology for health behavior change as well as identified similar barriers and a few of the technological requirements identified in our study. However, while the study explored risk behaviors like smoking and being overweight, the focus was not on supporting clinicians in oncology settings. The study participants were physiotherapists, nurses, nutritionists, and physicians, lacking oncologist participation. Moreover, the study primarily focused on health care professionals’ perception of patient needs when patients receive behavior change treatment and less on health care professionals’ needs to improve engagement with their patients. Our work expands on these findings by comprehensively exploring oncologists’ preferred roles and needs across the entire intervention life cycle, from patient referral to providing ongoing reinforcement.

The studies on clinicians’ perspectives of implementing exercise-based rehabilitation in a cancer unit [32,43] align more closely with our clinical context and include some oncologists in the study participants. Both aforementioned studies and our study emphasize the importance of streamlined referral processes and enhanced intervention visibility to clinicians. However, the 2 studies did not identify how oncologists wanted referral and reinforcement to be done, and the support oncologists require. Our research addresses this gap and advances the existing contributions by identifying oncologists’ preferences and support needs for referring patients to relevant interventions and reinforcing patients’ goals longitudinally. We proposed detailed information needs and specific technological strategies, such as using automated recommender systems for seamless referral and integrated EHR-based tools that provide just-in-time updates and conversation aids. A previous study [43] highlighted that continuous promotion of interventions to clinicians is essential, as staff turnover means new clinicians often join without knowledge of existing programs. Our study advances this narrative by identifying oncologists’ desire for an automated reminder system, integrated into the existing EHR workflows, that bridges the informational gaps and ensures both current and incoming clinicians receive timely, consistent updates about available interventions.

Ultimately, existing related literature has offered valuable insights into some of the clinicians’ perspectives and needs; however, our study extends this understanding by focusing on and providing a comprehensive examination of oncologists’ requirements throughout the health behavior change intervention life cycle. Our proposed detailed, low-burden, technologically enabled recommendations deliver novel insights that can inform the development of more effective and sustainable health behavior change interventions in cancer care.

### Limitations

This study’s scope focused primarily on gathering oncologists’ requirements and did not extend to evaluating the impact of the proposed support mechanisms on clinical workflows, workload, or burnout. It also did not include the perspectives of other cancer care team members, such as nurses, who might equally participate in holding behavior change conversations with patients and their family members. Additional implementation research is needed to assess the real-world implications of the proposed recommendations and ensure that they effectively address clinicians’ needs without inadvertently contributing to new challenges or unintended consequences. In addition, all oncologists were health care providers from 11 clinics within a National Cancer Institute–designated comprehensive cancer center that uses the Epic EHR messaging system. Focusing on these clinics might have closely connected our findings to the Epic system. While all study participants used Epic, using text-based EHR reports should be scalable and transferable.

Similarly, our study was situated within a hospital network located in an urban setting where EHRs are widely deployed and adopted. Therefore, our findings may not fully capture the perspectives of oncologists serving cancer survivors in rural or underserved areas or with very limited levels of digital health infrastructure. Nonetheless, we do not believe that our recommendations are limited to hospitals in urban settings, as we focused on recommendations that are lower in resources due to their ability to generalize and scale but may be adopted in medical settings where EHRs are deployed and used. Future research could explore perspectives across diverse health care settings, for example, examine how regional and demographic characteristics influence oncologists’ preferences and the design of interventions to better meet the needs of diverse patient populations.

Finally, while this study focuses on oncologists, PCPs have been recognized to play a significant role in cancer survivorship care [81,82]. However, PCPs also report known challenges and unmet requirements such as insufficient knowledge of cancer survivorship care, time constraints [41,83], and anxiety associated with delivering such care [83], which in many ways mirror the barriers identified by oncologists. Consequently, our design recommendations, although codeveloped with oncologists, could also help address these overlapping challenges faced by PCPs in delivering comprehensive survivorship care. Nonetheless, future research could further explore how to support PCPs, as part of the continuum of care, to contribute to implementing and reinforcing digital multiple health behavior change interventions, especially for patients whose care involves both oncologists and PCPs.

Despite these limitations, the findings in this study are strengthened by its purposive sampling approach, which aimed to capture the perspectives of clinicians with diverse expertise and experiences. The diverse expertise of the study participants across various cancer types and clinical specialties facilitated the process of cross-referencing, consistently comparing, and establishing the richness of the collected data. It also enhances the transferability and relevance of the findings to a broader range of oncology care settings and interventions targeting health behavior change among cancer survivors. Moreover, the multidisciplinary nature of the research team, comprising subject-domain experts across 4 relevant fields, provides a richer and more holistic context for the knowledge contributions made by this study. Finally, we adhered to the standards for reporting the qualitative research checklist [52], which is designed to enhance the transparency of qualitative studies by establishing clear reporting criteria for every aspect of the research process.

### Conclusions

This study captures oncologists’ perspectives on low-burden support to facilitate their roles in addressing risk behaviors and promoting health behavior change among cancer survivors. Our findings reveal that clinicians are motivated to engage in 2 key action-oriented communication pathways: referring patients to relevant interventions and reinforcing intervention goals. However, effectively performing these actions requires addressing specific informational and technological requirements articulated by clinicians themselves.

Crucially, oncologists emphasized the need for seamless integration of patient information and communication channels into existing EHR workflows. Clinicians emphasized the need for concise, text-based EHR updates on patient enrollment and progress, delivered just-in-time for clinical encounters, as well as specific language to guide patient conversations. In addition, our findings highlight the value of parallel systems, such as recommender systems for patient identification, EHR messaging with complementary print materials for referrals, and reminder systems to prompt timely discussions. By developing these low-burden, workflow-integrated solutions that facilitate timely communication and collaborative care, we can empower oncologists to play more active roles in promoting health behavior change. Future research should evaluate the impact of these proposed strategies on clinician and care team engagement, clinical workflow, and patient outcomes across diverse health care settings, ensuring that efforts to enhance integration with cancer care teams do not inadvertently contribute to increased burnout.

## Abbreviations

EHR: electronic health record
PCP: primary care physician

## Data Availability

The datasets generated or analyzed during this study are available from the corresponding author upon reasonable request.
